# Changes in biceps brachii muscle hardness assessed by a push-in meter and strain elastography after eccentric versus concentric contractions

**DOI:** 10.1038/s41598-022-13184-3

**Published:** 2022-06-02

**Authors:** Mitsuyoshi Murayama, Takayuki Inami, Norihiro Shima, Tsugutake Yoneda, Kazunori Nosaka

**Affiliations:** 1 Institute of Physical Education, Keio University, Address 4-1-1, Hiyoshi, Kouhoku-ku, Yokohama, 223-8521 Japan; 2 Department of Sport and Health Science, School of Sport and Health Science, Tokai Gakuen University, Aichi, Japan; 3 Department of Physiology, School of Health and Sports Science, Juntendo University, Chiba, Japan; 4 Centre for Human Performance, School of Medical and Health Sciences, Edith Cowan University, Joondalup, Australia

**Keywords:** Ultrasonography, Rehabilitation, Biomedical engineering

## Abstract

Changes in biceps brachii muscle hardness assessed by a push-in meter (PM) and strain elastography (SE) were compared between eccentric (ECC) and concentric contractions (CON) of the elbow flexors to test the hypothesis that muscle hardness would increase greater after ECC. Ten men performed 5 sets of 10 ECC with their non-dominant arms and 5 sets of 10 CON with their dominant arms using a dumbbell corresponding to 50% of maximum voluntary isometric contraction (MVIC) force at 90º elbow flexion. Before and 1–4 days after the exercise, MVIC force, elbow joint angles, upper-arm circumference, and muscle soreness as muscle damage makers, and biceps brachii muscle hardness at maximally extended elbow joint by PM and SE were measured. Changes in these measures over time were compared between ECC and CON. All muscle damage markers showed greater changes after ECC than CON (*p* < 0.001). Muscle hardness assessed by PM and SE increased (*p* < 0.05) and peaked at 4 days post-ECC with 154.4 ± 90.0% (PM) and 156.2 ± 64.2% (SE) increases from the baseline, but did not change significantly after CON. The changes in muscle hardness post-ECC were correlated between PM and SE (r = 0.752, *p* < 0.001). A correlation (*p* < 0.001) between the normalized changes in resting elbow joint angle and changes in muscle hardness assessed by PM (r = − 0.772) or SE (r = − 0.745) was also found. These results supported the hypothesis and suggest that the increases in muscle hardness after ECC were associated with muscle damage (increased muscle stiffness), and PM and SE detected muscle hardness changes similarly.

## Introduction

Muscle hardness is defined as the resistance of muscle tissue (responded force) against deformation applied to the muscle^1^. Muscle hardness is affected by several physiological factors such as passive and active tension of muscle^2^ and intramuscular pressure^3^. Increases in muscle hardness have been reported after eccentric exercise^1,4,5^ and a full marathon^6^ that induced muscle damage. For example, our previous study^1^ showed that biceps brachii muscle hardness assessed by a push-in meter (PM) increased 30–100% from baseline at 1–5 days after maximal eccentric exercise of the elbow flexors.

It is well known that high intensity eccentric exercise induces muscle damage that is represented by decreased muscle strength, reduced range of motion (ROM), muscle swelling and delayed onset muscle soreness^7^. The increase in muscle hardness after eccentric exercise-induced muscle damage was considered to be affected by decreases in ROM and increases in circumference due to swelling^1^. Although it is less significant than eccentric exercise, concentric and isometric exercises also induce muscle damage^8^. Concentric exercise increases muscle oxygenation and total hemoglobin volume, enhances vasodilatory action, and increases blood flow greater than eccentric exercise^9,10^. Vasodilation and muscle pumping induced by muscle contractions lead to exercise hyperemia^11^. Therefore, it is possible that muscle hardness is also increased after concentric exercise. However, no previous study has compared muscle hardness changes over days after eccentric versus concentric exercise.

To assess muscle hardness, some of the previous studies used a PM in which a probe was pushed to a muscle from the body surface to evaluate the relationship between displacement and responded force^1,12^. We have developed a PM method to assess muscle hardness, and examined its validity against ultrasound strain elastography (SE)^13^. SE images the strain distribution generated by compression of an ultrasonic probe using changes in ultrasonic signals, and provides strain distribution in any region of interest (ROI)^14^. We have reported significant correlations between the Young’s modulus assessed by PM and Young’s modulus converted from strain ratio (SR) from SE in resting and contracting muscles^13^. This suggested that muscle hardness values obtained by PM and SE were comparable. However, it is not known whether the changes in muscle hardness after eccentric or concentric exercise can be detected similarly by PM and SE.

Muscles become stiffer and harder after exercise, which is felt subjectively when the affected muscles are stretched and/or palpated. To quantify muscle hardness changes, PM and SE are often used. However, it is not known whether the changes in muscle hardness values obtained from PM and SE are similar, and how they respond to eccentric and concentric exercises. Thus, it is important to clarify how the two muscle hardness measures respond to eccentric or concentric exercise to better understand muscle hardness changes after exercise.

The aim of the present study, therefore was to compare biceps brachii muscle hardness changes after eccentric versus concentric contractions of the elbow flexors using PM and SE. We hypothesized that muscle hardness would increase greater after eccentric than concentric exercise, and muscle hardness changes assessed by PM and SE would be comparable.

## Methods

### Participants

Ten healthy men (average ± SD age: 25.8 ± 3.9 years, height: 180.0 ± 7.7 cm, body mass: 77.6 ± 11.2 kg) participated in the present study. The sample size was estimated by a priori power analysis using the G ∗ Power software (Version 3.1.9.2, Universität Kiel, Germany)^15^. We obtained the effect size of 0.4 based on the previous study^16^ in which eccentric and concentric exercises of the elbow flexors were compared for changes in some muscle damage markers. We calculated the sample size for the present study based on the effect size with a significance level (α) of 0.05 and power (1-β) of 0.80. It was estimated that at least 8 participants per group were necessary. Since the present study utilized an arm-to-arm comparison model, 10 participants were recruited, considering a possible smaller effect size than 0.4 for some variables. The participants had not performed any upper arm resistance training in the last 6 months and had no musculoskeletal injury of the upper limb. The study was approved by the Human Ethics Committees of Keio University (Japan) and Edith Cowan University (Australia). All participants were informed of the purpose, examination procedures and the potential risk of the study, and signed an informed consent. All procedures were carried out in accordance with the Declaration of Helsinki.

### Study design

**Figure 1:**
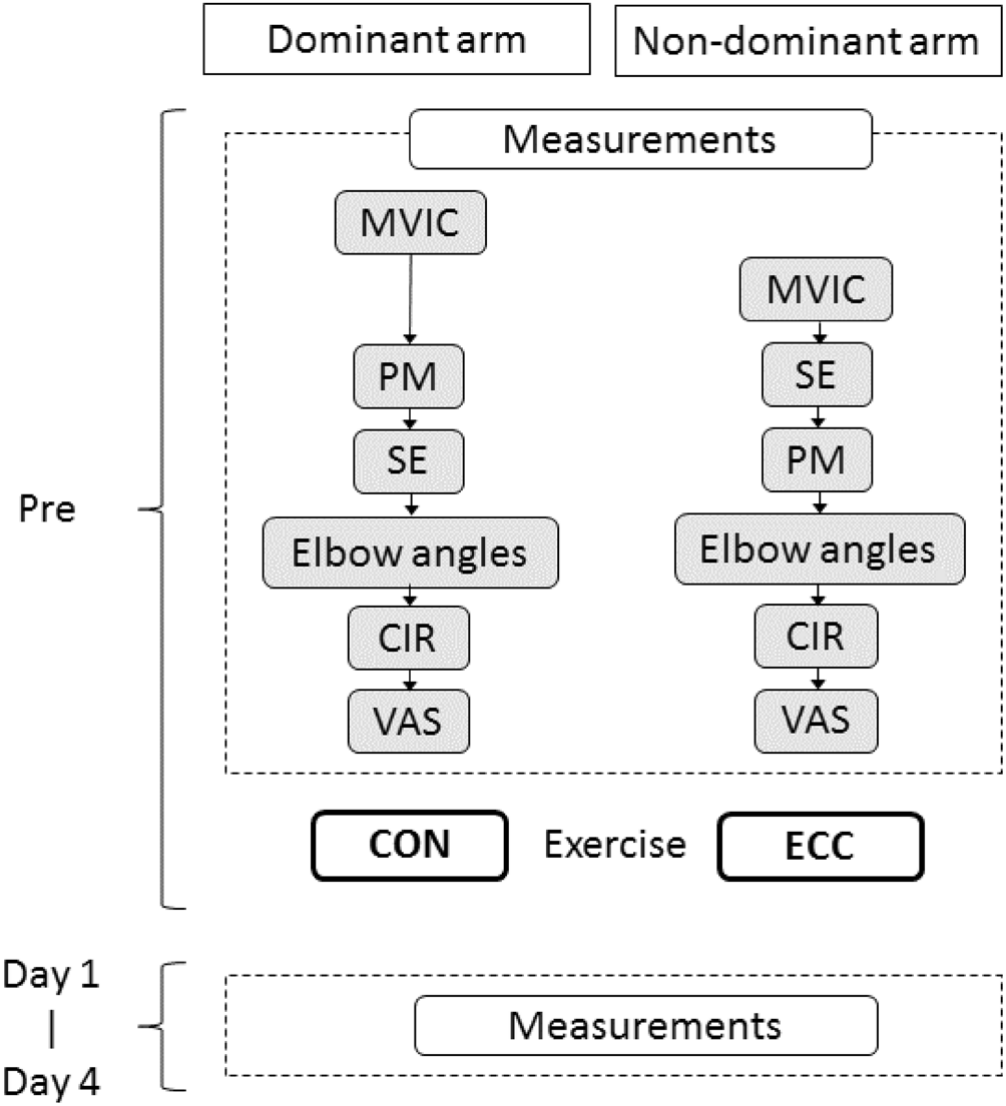
Schematic representation of the study protocol. The assessments of muscle damage markers and muscle hardness in the dominant and non-dominant arm were conducted sequentially as shown in the “Measurements” that consisted of maximal voluntary isometric contraction force (MVIC), push-in meter (PM), strain elastography (SE), relaxed, extended and flexed elbow angle (Elbow angles), upper arm circumference (CIR) and visual analog scale (VAS) for muscle soreness, before (Pre) and 1–4 days after exercise (Day 1- Day 4). After the Pre assessments, concentric (CON) and eccentric contractions (ECC) were performed by the dominant and non-dominant arm, respectively.

We used an arm-to-arm comparison model: one arm performed eccentric exercise, and the other arm performed concentric exercise. Thus, the independent variable of the present study was muscle contraction (action) type. The dependent variables included muscle hardness and muscle damage measures that were taken before (Pre), and 1, 2, 3, and 4 days after exercise from each arm (Fig. 1). The post-exercise measurements were taken at a similar time of the day to that of the exercise with the difference being no more than two hours between days. Changes in the measures over time were compared between arms that performed eccentric exercise versus concentric exercise.

### Exercise

Each participant sat on an arm curl bench and performed 5 sets of 10 eccentric contractions (ECC) with the non-dominant arm and 5 sets of 10 concentric contractions (CON) with the other arm using a dumbbell set at 50% of their maximal voluntary isometric contraction (MVIC) force for each arm^16^. The non-dominant arm lowered the dumbbell from an elbow flexed (90°) to a fully extended position (0° = full extension) in 3–4 s, and the dominant arm lifted the dumbbell from the extended position to the flexed position in 3–4 s alternatively. The reason for the partial range of motion was that the load to the elbow flexors is reduced at the angles exceeding 90° (e.g., at 120° elbow flexion, the load of the dumbbell was minimum to the elbow flexors) in the preacher curl exercise. The non-dominant arm was used for the eccentric exercise to minimize possible inconvenience in daily activities of the participants, because it has been known that eccentric exercise induced greater symptoms of muscle damage such as decreases in muscle function and joint range of motion, and muscle pain than concentric exercise^17^. The transfer of the dumbbell from one arm to the other was assisted by the investigator^16^. Thus, each contraction was repeated every 5 s or so, and a 2-min rest was inserted between sets. Newton et al.^18^ did not find any significant difference between the dominant and non-dominant arms for their responses to maximal eccentric exercise. Thus, it seems unlikely that the use of the dominant arm for eccentric exercise was a problem.

### Muscle hardness assessment

**Figure 2:**
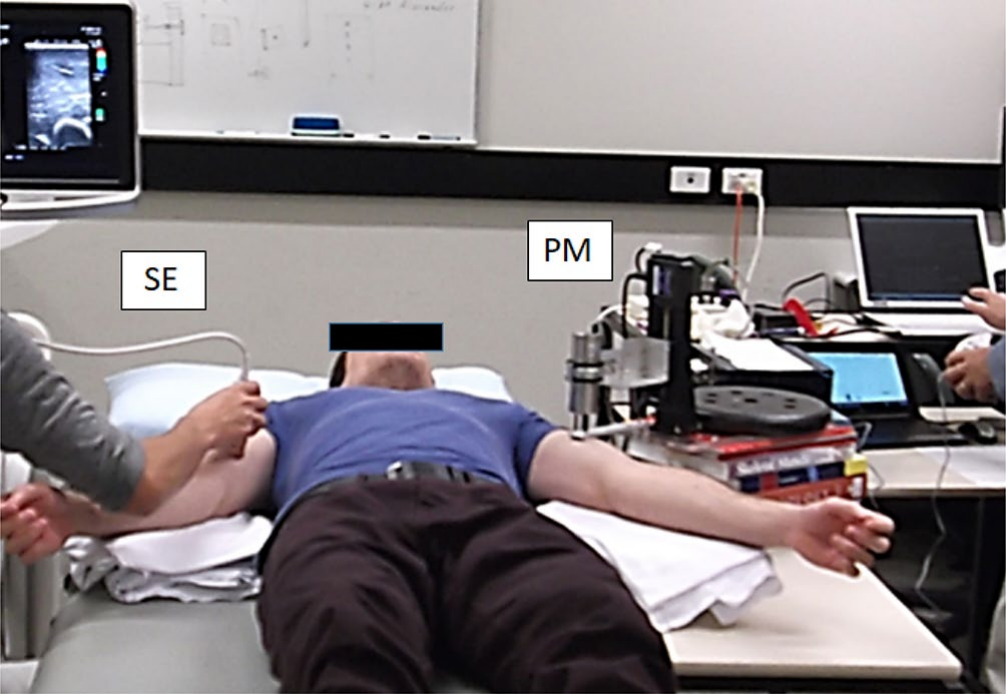
Muscle hardness assessment. Biceps brachii muscle hardness of both arms was assessed by a push-in meter (PM) and strain elastography (SE) simultaneously. In this example, SE for the right arm, and PM for the left arm. (Microsoft Powerpoint 2019, https://www.microsoft.com/ja-jp/microsoft-365/powerpoint).

Each participant lay on his back on a massage table with relaxing both arms. B-mode ultrasound images (Prosound F75; Hitachi Aloka Medical, Japan) were taken from the point where the biceps brachii muscle thickness was the largest, and the sites on both sides were marked by a semi-permanent ink pen. Muscle hardness measurements by a push-in meter (PM) and ultrasound strain elastography (SE) were taken in resting condition from the same site over time. PM and SE measurements were performed concurrently but one arm for PM and the other arm for SE by the different examiners (Fig. 2). It is known that the elbow joint angle for extension is decreased and that for flexion is increased after eccentric exercise of the elbow flexors when muscle damage is induced^19^. The elbow joint angle could become more flexed even in a resting state after eccentric exercise, thus the measurement of muscle hardness was made with both a relaxed elbow position and a passively extended elbow position^1^. The relaxed position was defined as a state in which the participant relaxed the arm and place it side of the body with its palm being placed up while lying on a bed. In the relaxed position, the forearm was supported using a towel placing under the forearm to eliminate the tension generated by the forearm. In the extended elbow joint measurement, the investigator extended the participant’s elbow joint forcibly to full extension (elbow joint angle was close to 0°) by holding his wrist and shoulder. However, in the comparison between ECC and CON, since the relaxed elbow joint angles were different among participants and affected by the exercises differently, the fully extended elbow joint position was used.

#### Push-in meter (PM)

**Figure 3:**
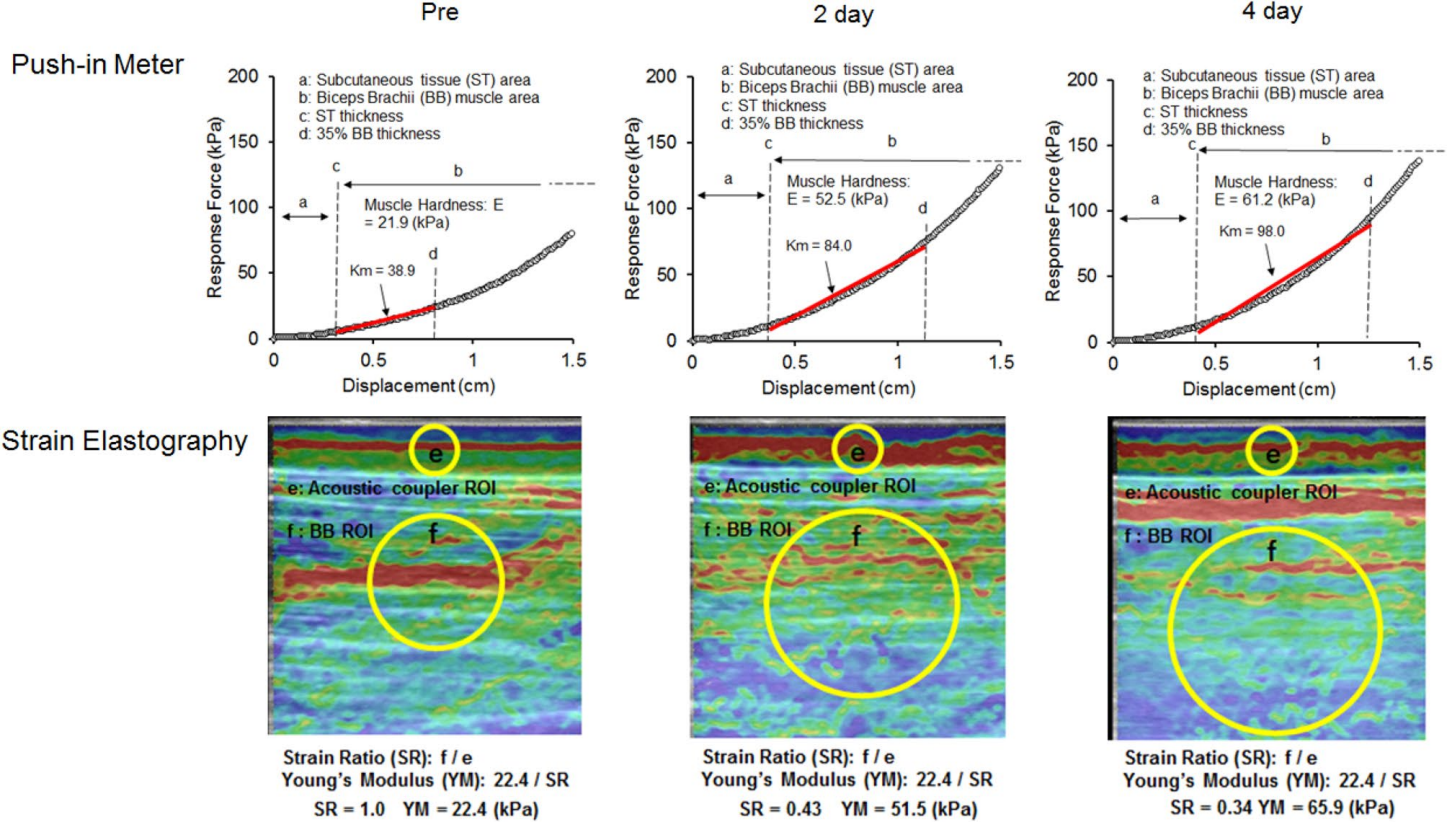
Typical examples of the force displacement relationships measured by push-in meter (PM, upper) and elastography images obtained by strain elastography (SE, lower) for biceps brachii (BB) muscle hardness assessment at before, 2 and 4 days after eccentric exercise. In PM, the force displacement relationship was divided into the subcutaneous area (**a**) and BB area (**b**), then muscle hardness value E was calculated using the slope (Km) of the force curve ranged in 0–35% BB thickness (from **c** to **d**). In SE, the region of interest (ROI) was set for acoustic coupler area (**e**) and a circle including the whole BB (**f**). Using a built-in software, strain ratio (SR) was calculated for each image as a ratio of strain of the muscle (**f**) divided by the strain of the acoustic coupler (e), then SR was converted to Young’s Modulus from the known Young’s modulus of the coupler (22.6 kPa) by the formula; 22.6/SR. (Microsoft Powerpoint 2019, https://www.microsoft.com/ja-jp/microsoft-365/powerpoint).

Muscle hardness was assessed using the force–displacement relationship which was recorded by a PM system (TK-HS100, Tokushu-keisoku, Japan). The PM system was described in detail in the previously published papers^13,20^. Briefly, based on a two-layered spring model by Horikawa et al.^12^, the relation between the depth of deformation (displacement) and the reaction force was divided into subcutaneous and muscle component based on the subcutaneous tissue thickness measured by a B-mode ultrasound. The probe angle in the PM assessment was always set vertical to the muscle^20^, thus the effect of the subcutaneous tissue thickness on the muscle harness measures was minimum. The muscle hardness value (E) was calculated by the following equation; E = Id (1-μ^2^) Km, where I is an influence coefficient, d is the diameter of the probe, μ is Poisson’s ratio and Km is the slope of the muscle component obtained from the force–displacement relationship. Therefore, E is depended on Km; however, since the force–displacement relationship shows a non-linearity that increases as indentation goes deeper, Km overestimation is likely to occur. To avoid this, the individual muscle thickness was considered. We proposed that the range for Km calculation in the force–displacement curve was normalized by the individual muscle thickness within 40% muscle thickness^20^. In the present study, E was calculated using the slope of the force curve ranged in 0–35% of a biceps brachii muscle thickness of each participant. The typical samples of the force–displacement relationship and Km slope are shown in Fig. 3.

#### Strain elastography (SE)

A diagnostic ultrasound system with elastography function (Prosound F75; Hitachi Aloka Medical, Japan) was used. SE evaluates the stress–strain relationship for the tissue hardness based on Hooke’s law applying the compression by the ultrasound transducer. The investigator placed the transducer along the longitudinal axis of muscle with the measurement point on the biceps brachii mid-belly in the centre and manually applied rhythmical compression-relaxation cycles to the muscle with consistent pressure. It was ensured that the transducer was pressed to the strain level of 2 or 3 while monitoring the level shown in the system screen, and the transducer angle was always perpendicular to the muscle belly, where as indicated by a mark on the skin^13^. Elastograph images were recorded while gently pressing a transducer with a reference material (acoustic coupler, Young’s modulus = 22.6 kPa: EZU-TECPL1, Hitachi Aloka Medical Japan). A circular region of interest (ROI) was set in the coupler and the muscle under the mark on the skin. Since previous studies have adopted ROI that occupies a large area of a target muscle^5,21^, the present study also set a ROI for the entire biceps brachii as shown in Fig. 3. Using a built-in software, strain ratio (SR) was calculated for each image as a ratio of strain of the muscle divided by the strain of the acoustic coupler. Therefore, SR shows muscle hardness from the relative relationship with the coupler. However, if the muscle is harder, SR value is smaller like a fractional function and it shows a non-linear relationship. Despite the fact that E was calculated in Young’s modulus, this numerical characteristic of SR complicates the comparison between the two. Therefore, the SR values by the SE measures were converted to Young’s modulus from the known Young’s modulus of the coupler by the formula; 22.6 / SR^13^.

### Muscle damage markers

The indirect markers of muscle damage consisted of MVIC force, elbow joint angles (flexed elbow angle: FANG, relaxed elbow angle: RANG and extended elbow angle: EANG) and range of motion (ROM), upper arm circumference (CIR) and muscle soreness. All criterion measures were assessed before (pre) and 1–4 days after exercise for each arm: ECC and CON respectively.

#### MVIC force

Muscle force of the elbow flexors of each arm was measured at 90° flexion. Each participant sat on a chair and the upper arm was placed on a table in a supination position, and a strap connected to a loadcell (LUR-A-SA1, Kyowa, Japan) was placed to the wrist. After two practical attempts to generate submaximal force, the participant was asked to generate maximal force for 3 s for each trial and repeated this 3 times with a 1-min rest between trials for each arm. The maximum force of the 3 trials was used as MVIC. The output force captured by the load cell was recorded to a computer (HP pavilion 15, HP Japan Inc., Japan) via a strain amplifier (AS2103, NEC Sanei, Japan) which was connected to a PowerLab system (ADInstruments, Australia) operated by a LabChart software (ADInstruments, Australia) installed^13^.

#### Elbow joint angles and ROM

While each participant was standing, the elbow joint angles in relaxed (RANG) and the maximal voluntarily flexed (FANG) and extended (EANG) positions of each arm were measured by a plastic goniometer manually. The landmarks of the measurement were marked on the skin by a semi-permanent ink pen. ROM of the elbow joint was determined as the difference between FANG and EANG. Three measurements were taken for each angle, and the mean of the three measurements was used to calculate ROM^22^.

#### Upper arm circumference (CIR)

While each participant was standing, relaxing and the arms were hanging down by his side, CIR was measured of the mid-portion of the upper arm by a tape measure for each arm. The landmark of the measurement was marked on the skin by a semi-permanent ink pen. The measurements were taken three times by the same investigator, and the mean of the three measures was used for further analysis^22^.

#### Muscle thickness

The thickness of the biceps brachii muscle was measured from a B-mode ultrasound image of SE for measuring muscle hardness. Therefore, muscle thickness of biceps brachii was measured in the extended elbow position for both arms.

#### Muscle soreness

The level of muscle pain of the upper arm was assessed by a visual analogue scale (VAS) with a 100-mm line anchoring 0-mm being no pain and 100-mm being the worst imaginable pain. The participant was asked to mark the level of perceived soreness on the VAS, when the elbow flexors were palpated by the investigator. The palpation was provided by the well-trained investigator who had practiced to apply the same pressure to different participants at different time points. The protocol was kept as consistent as possible between days and among participants, and all measurements were taken by the same investigator throughout the study. The investigator placed his index and middle fingers over the participant’s biceps brachii muscle (at 3, 8, 13 cm above the elbow crease), brachialis muscle and brachioradialis muscle and applied pressure with the tips of the finger toward the deeper tissues for approximately 3 s. The average value of VAS for 5 muscle points was used as muscle soreness in palpation. The landmark of the measurement was marked on your skin by a semi-permanent ink pen and the measurements were taken by the same investigator throughout the study^22^.

### Statistical analyses

The normality and the homogeneity of the data were verified by the Shapiro–Wilk test and the Levene test, respectively. A two-way repeated measure analysis of variance (ANOVA) was used to compare between ECC and CON for changes in the muscle damage markers (MVIC, CIR, FANG, RANG, EANG, ROM and muscle soreness VAS) and muscle hardness assessed by PM and SE over time. Dunnet’s post-hoc test was performed for analysis of difference from baseline for the above variables. The pre-exercise baseline values were considered as the control, and the Dunnet's test compared it with the values obtained at 1–4 days after exercise. Pearson’s correlation analyses were performed between PM and SE regarding muscle hardness measurement, and between muscle damage makers and each muscle hardness assessed by PM and SE. SPSS version 27.0 was used to perform these statistical analyses. A significance level was set at *p* < 0.05. All values are expressed as mean ± standard deviation (SD).

### Ethics approval

All procedures performed in the present study were approved by the local Institutional Ethics Review Board (Keio University and Edith Cowan University), and in accordance with the 1964 Helsinki declaration and its later amendments. The informed consent was obtained from all participants included in the study.

## Results

### Changes in muscle damage markers

**Figure 4:**
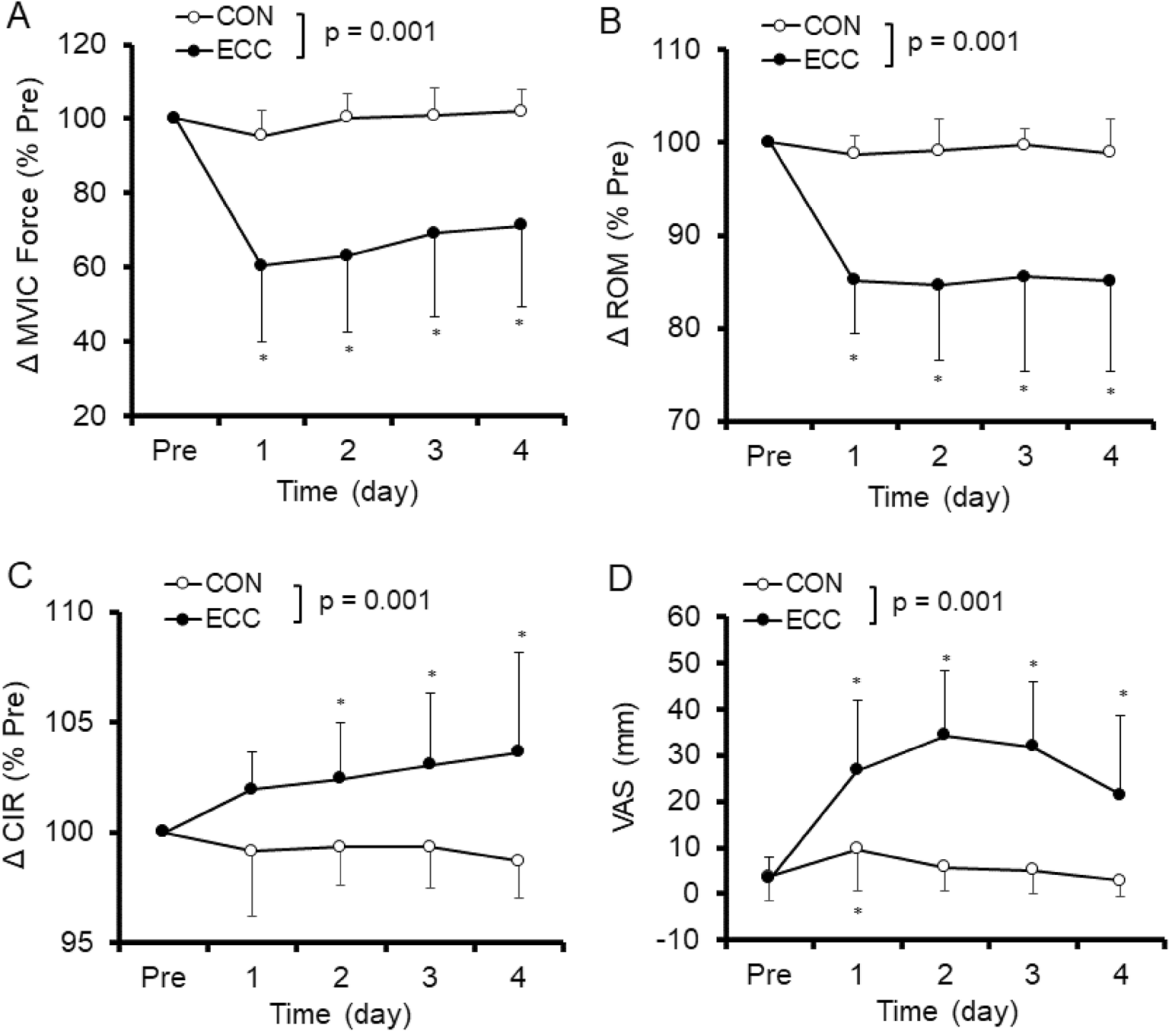
Normalized changes (mean ± SD) in maximal voluntary isometric contraction (MVIC) force (**A**), range of motion (ROM, **B**), upper arm circumference (CIR, **C**) and muscle soreness assessed by a 100-mm visual analogue scale (VAS, **D**) before (Pre) and 1–4 day after exercise. A significant (*p* < 0.001) interaction effect was found between eccentric (ECC) and concentric (CON) conditions for all variables. * shows significant (*p* < 0.05) difference from the baseline value.

No significant differences in the baseline values of any of the muscle damage markers were evident between arms. A significant condition (ECC vs CON) x time interaction effect was evident in the absolute values for MVIC force (F = 23.07, p = 0.001), FANG (F = 22.55, p = 0.001), RANG (F = 5.4, p = 0.002), EANG (F = 3.02, p = 0.03), ROM (F = 10.01, p = 0.001), CIR (F = 6.16, p = 0.001), and VAS (F = 12.89, p = 0.001). Similarly, significant interaction effects were observed in all variables except VAS for the relative values to baseline. Figure 4A–C show relative changes in MVIC force, ROM and CIR from baseline, respectively, and Fig. 4D shows changes in absolute value of VAS before and 1–4 day after ECC and CON. MVIC force decreased (*p* < 0.05) 39.7 ± 20.6% from 323.1 ± 81.4 N (baseline) to 207.2 ± 118.1 N (1 day post-ECC), and remained lower than the baseline (*p* < 0.05) for 4 days post-ECC. On the other hand, MVIC force slightly decreased at 1 day and returned to the baseline by 2 days after CON. ROM decreased (*p* < 0.05) 14.8 ± 5.7% from 137.5 ± 5.8° (baseline) to 117.0 ± 8.1° (1 day post-ECC) and remained lower than the baseline (*p* < 0.05) for 4 days after ECC. However, no significant changes in ROM were observed after CON. CIR increased (*p* < 0.05) 2.4 ± 2.5% from 290.5 ± 31.9 mm (baseline) to 297.1 ± 28.4 mm (2 days post-ECC) and remained higher than the baseline (*p* < 0.05) for 4 days post-ECC. In contrast, no significant changes in CIR were evident after CON. VAS increased after ECC from baseline (3.2 ± 4.8 mm) and peaked at 2 days post-exercise (34.3 ± 14.0 mm, *p* < 0.05). For CON, VAS slightly increased (*p* < 0.05) from baseline (3.7 ± 5.2 mm) to 1 day post-exercise only (9.6 ± 9.2 mm).

**Figure 5:**
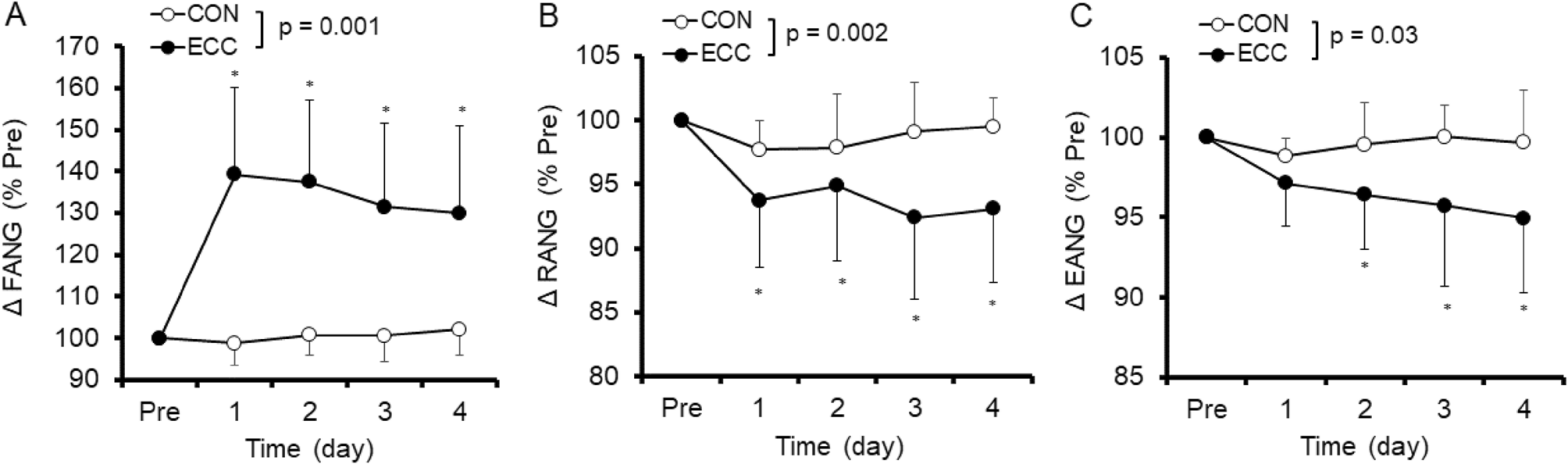
Normalized changes (mean ± SD) in flexed elbow angle (FANG, **A**), relaxed elbow angle (RANG, **B**) and extended elbow angle (EANG, **C**) before (Pre) and 1–4 day after exercise. A significant (*p* < 0.05) interaction effect was found between eccentric (ECC) and concentric (CON) conditions for FANG (*p* < 0.001), RANG (*p* < 0.01) and EANG (*p* < 0.05). * shows a significant (*p* < 0.05) difference from the baseline value.

Figure 5 shows relative changes in FANG, RANG and EANG from baseline. FANG increased (*p* < 0.05) 39.3 ± 20.8% from 42.0 ± 8.0° (baseline) to 57.3 ± 7.0° (1 day post-ECC) and remained higher than baseline (*p* < 0.05) for 4 days post-ECC. On the other hand, no significant changes in FANG were observed after CON. RANG decreased (*p* < 0.05) 2.3 ± 2.3% from 170.0 ± 7.3° (baseline) to 157.1 ± 13.5° (4 days post-ECC), but no significant changes were observed after CON. EANG also decreased (*p* < 0.05) 5.1 ± 4.7% from 179.5 ± 6.3° (baseline) to 170.5 ± 11.6° (4 days post-ECC), but no significant changes were observed after CON.

### Changes in muscle hardness after eccentric and concentric exercise

**Figure 6:**
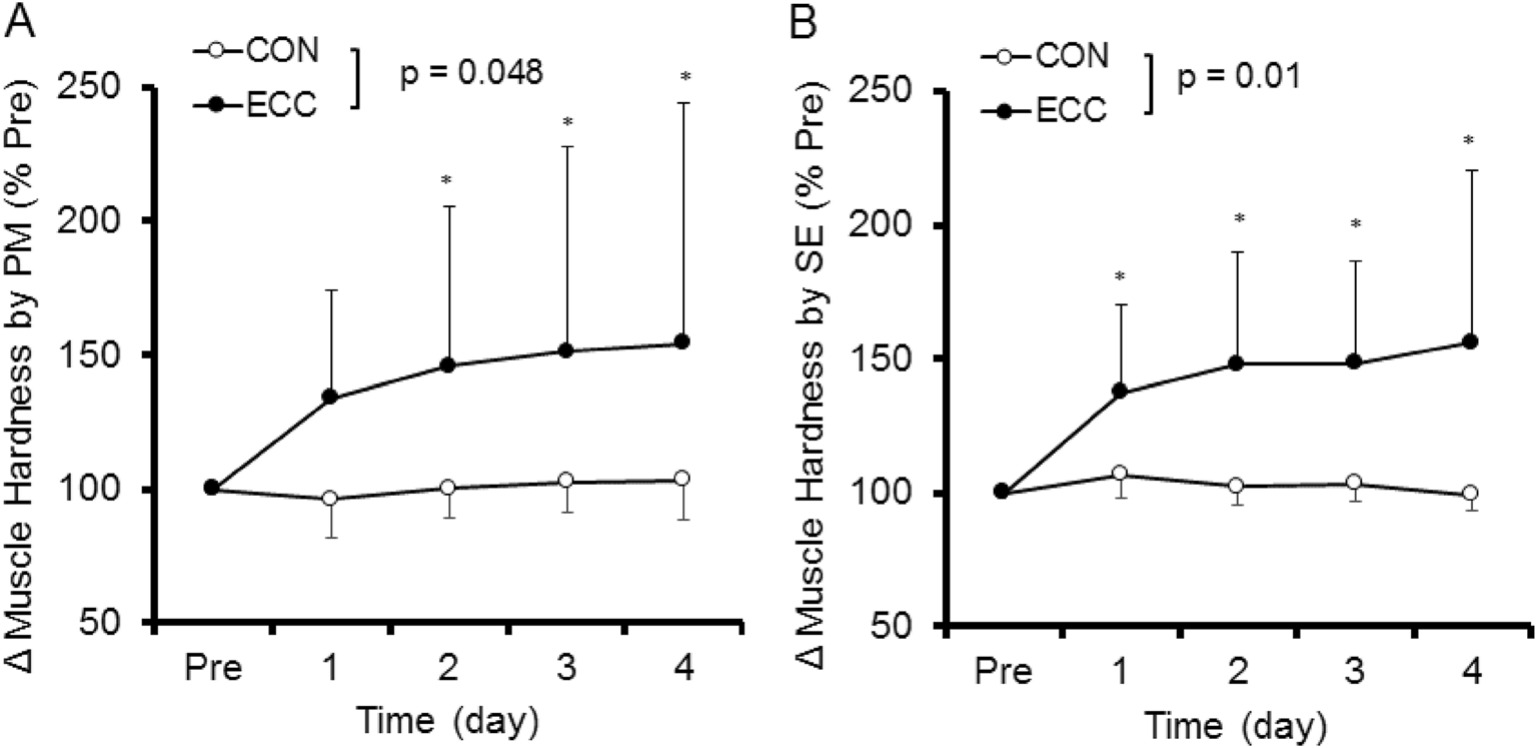
Normalized changes (mean ± SD) in muscle hardness measured by push-in meter (PM, **A**) and strain elastography (SE, **B**) before (Pre) and 1–4 day after exercise. A significant interaction effect was found between eccentric (ECC) and concentric (CON) conditions for changes in PM (*p* < 0.05) and USE (*p* < 0.01). * shows a significant (p < 0.05) difference from the baseline value.

Muscle hardness assessment was performed at relaxed and extended elbow positions. However, it was considered inappropriate to compare the muscle hardness in the relaxed position between ECC and CON, because there was no change in the joint angle after CON. Therefore, a comparison between ECC and CON was made for the extended elbow position only. No significant differences in the baseline values of the muscle hardness assessed by PM and SE were evident between arms. A significant condition (ECC vs CON) x time interaction effect was evident for absolute changes in muscle hardness assessed by PM (F = 3.95, p = 0.048) and SE (F = 6.44, p = 0.01). Similarly, significant interaction effects were observed in PM and SE for the relative changes from the baseline values. Figure 6A and B show changes in muscle hardness assessed by PM and SE, respectively, expressed as percent changes from baseline. Muscle hardness (in Young’s module) assessed by PM and SE increased (*p* < 0.05) from 26.1 ± 8.5 kPa to 37.6 ± 16.5 kPa (54.4 ± 89.5%) and from 29.3 ± 11.6 kPa to 41.7 ± 11.6 kPa (56.2 ± 64.2%), respectively from baseline to 4 days post-ECC. On the other hand, muscle hardness assessed by PM and SE did not show significant changes from the baseline value (20.2 ± 7.0 kPa) after CON.

**Figure 7:**
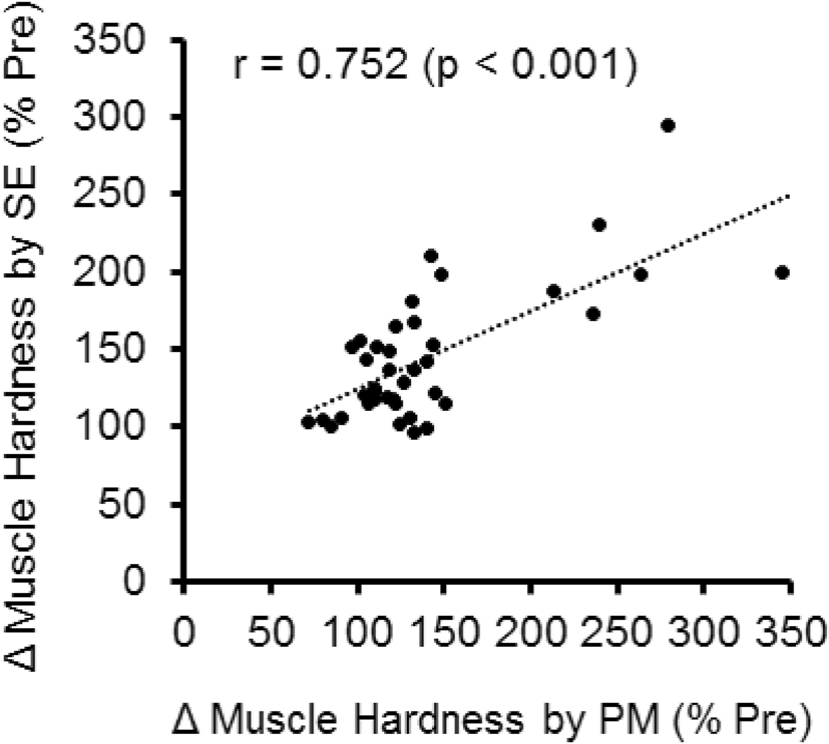
Relationship between normalized muscle hardness assessed by push-in meter (PM) and that by strain elastography (SE). A significant correlation was evident (r = 0.752, p < 0.001, regression equation: y = 0.5x + 74.0).

Figure 7 shows the relationship between PM and SE measures for normalized muscle hardness changes. A significant strong correlation was evident between the two measures (r = 0.752, *p* < 0.001, regression equation: y = 0.5x + 74.0).

### Relationship between muscle damage and muscle hardness after eccentric exercise

**Figure 8:**
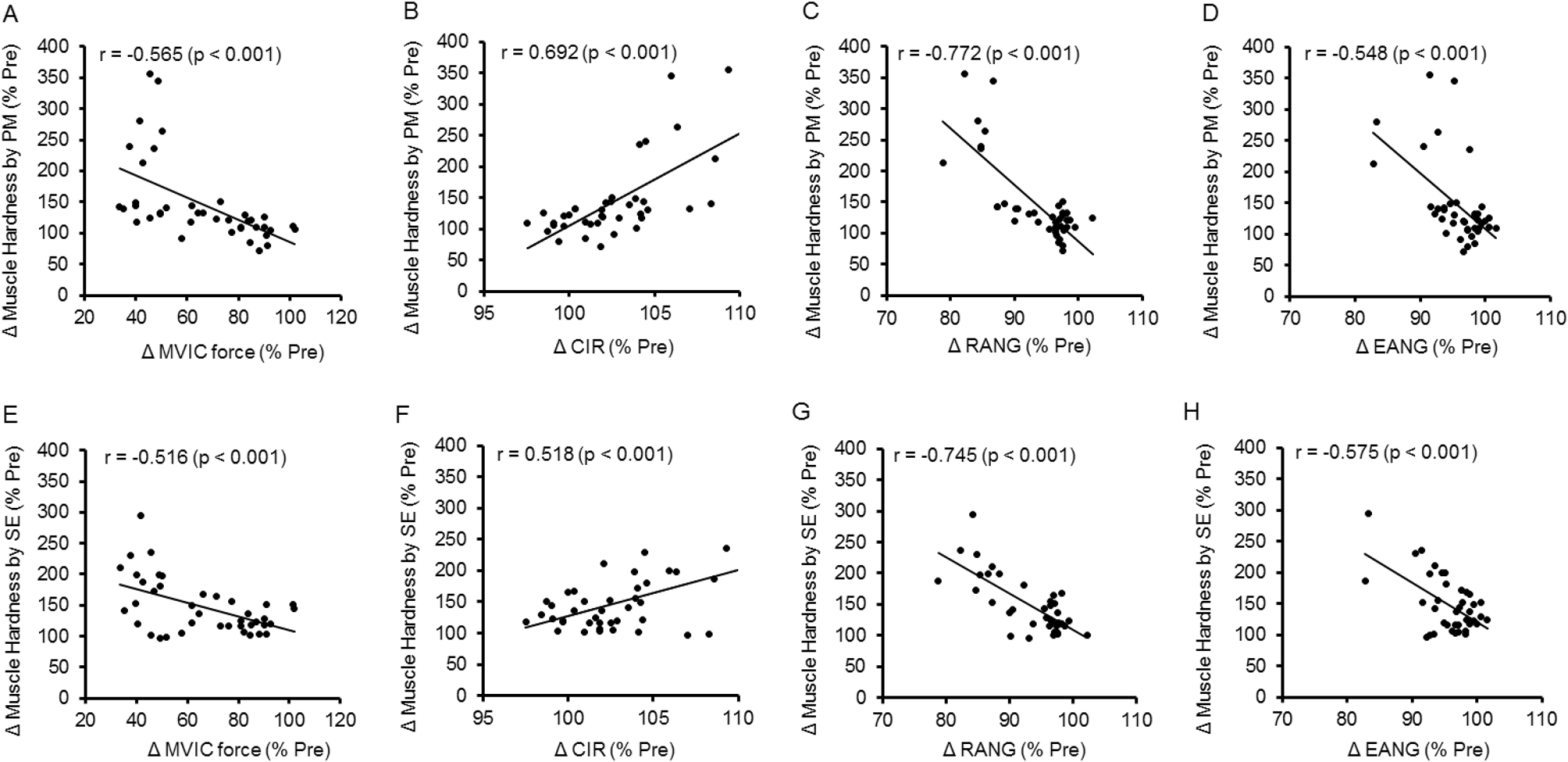
Relationship between muscle damage variables (maximal voluntary isometric contraction (MVIC) force, circumference (CIR), relaxed elbow angle (RANG) and extended elbow angle (EANG)) and muscle hardness assessed by push-in meter (PM) and strain elastography (SE). A significant (p < 0.001) correlation was evident between MVIC and PM (r = − 0.565, **A**), MVIC and SE (r = − 0.516, **E**), CIR and PM (r = 0.692, **B**), CIR and SE (r = 0.518, **F**), RANG and PM (r = − 0.772, **C**), RANG and SE (r = − 0.745, **G**), EANG and PM (r = − 0.548, **D**) and EANG and SE (r = − 0.575, **H**).

Figure 8 shows the relationship between normalized changes in muscle damage markers (MVIC force, CIR, RANG and EANG) and normalized changes in muscle hardness assessed by PM and SE following ECC. The damage marker that showed the highest (strong) correlation with muscle hardness was RANG (*p* < 0.001), with a correlation coefficient of − 0.772 for PM (Fig. 8C) and − 0.745 for SE (Fig. 8G). MVIC force and EANG also showed significant negative moderate correlations with muscle hardness assessed by PM (r = − 0.565: Fig. 8A and r = − 0.548: Fig. 8D, respectively) and by SE (r = − 0.516: Fig. 8E and r = − 0.575: Fig. 8H, respectively). CIR also showed a significant correlation with muscle hardness assessed by PM (r = 0.692, strong: Fig. 8B) and by SE (r = 0.518, moderate: Fig. 8F).

## Discussion

To the best of our knowledge, this was the first study to compare changes in muscle hardness between ECC and CON using PM and SE. Our hypothesis was that muscle hardness would increase greater after ECC, since greater muscle damage is induced by ECC than CON. As shown in Figs. 4 and 5, significant changes in muscle damage markers such as decreases in RANG, EANG and ROM, and increases in FANG, CIR and VAS were observed only after ECC, and the magnitude of decreases in MVIC force was greater after ECC than CON. These results suggested that only ECC induced muscle damage, which was in line with the previous studies showing eccentric exercise induced greater muscle damage and inflammation than concentric exercise^16,17^. As shown in Fig. 6, muscle hardness assessed by PM and SE increased only after ECC, and the PM and SE showed similar changes in muscle hardness. Thus, the hypothesis was supported by the results, and it was also confirmed that PM and SE provide comparable information about muscle hardness changes.

We previously reported that significant correlations between PM and SE in the resting (r = 0.615) and contracting biceps brachii muscle (r = 0.768)^13^. The present study showed that the normalized changes in muscle hardness assessed by PM and SE measures were significantly correlated (r = 0.752) (Fig. 7). The time course of the changes was also similar between the two measures (Fig. 6). These suggest that PM and SE reflect the same aspects of changes in the muscle, although what is actually measured by PM (force) is different from that in SE (strain). The compression of the ultrasonic probe in SE was only a few mm along the long axis of the muscle over the reference coupler, but the PM probe (10 mm in diameter) was pushed in a depth of 20 mm to the muscle. Therefore, PM measured muscle hardness from a real force–displacement relationship, but SE measured the relative strain in ROI compared to the reference coupler. However, it was possible to convert muscle reaction force measured by PM and strain measured by SE to Young’s modulus and compare them in the same unit, because the Young’s modulus can be obtained based on the force–displacement relationship in the PM measure, and strain obtained from the SE can be calculated as a ratio to the known Young’s modulus of the reference coupler (22.4 kPa)^13^.

As shown in Fig. 6, muscle hardness increased from pre- to 1 day post-ECC, and increased further between 1 and 4 days after ECC. This change pattern was similar to that of upper arm circumference (Fig. 4C), FANG (Fig. 5A) and EANG (Fig. 5C). Moreover, the normalized changes in CIR, RANG and EANG were significantly correlated with the normalized changes in muscle hardness following ECC, and the highest correlation was evident between RANG and muscle hardness assessed by PM (r = − 0.772) or SE (r = − 0.745) (Fig. 8). These suggest that the factors changing the RANG overlap with the factors changing muscle hardness. We have demonstrated an increase (100%) in muscle hardness in the extended elbow joint position from 1 to 3 days after ECC together with a decrease in ROM^1^. In the present study, the muscle hardness increased up to 4 days post-ECC but the magnitude of the increase was smaller (PM: 54.4%, SE: 56.2%) than that (PM: 100%) of the previous study^1^. However, the pattern of increase in muscle hardness was similar to the time course of decrease in RANG and EANG in both studies. Niitsu et al.^4^ also reported 30% increase in muscle hardness after 45 eccentric contractions of the elbow flexors. It has been documented that mechanical disruption of sarcomeres induced by eccentric contractions increases myoplasmic Ca^2+^, which increases passive tension and swelling^23^. Muscle hardness showed a high correlation with active and passive muscle tension^2^. It is considered that the decrease in RANG was most associated with increased passive tension after muscle damage. This may explain the high correlation between RANG and muscle hardness for their changes.

As shown in Fig. 8B and F, a significant correlation between muscle hardness and muscle swelling was also evident. Our previous study also showed a delayed increase in muscle hardness in the relaxed elbow joint position at 4 to 5 days after exercise when increased CIR was the largest^1^. It was possible that the increase in internal pressure due to swelling was associated with the increase in muscle hardness. Howell et al.^24^ reported that an increased tissue volume limited the stretching of connective tissue elements of the perimysium and/or epimysium, and Cheleboun et al.^25^ stated that swelling was associated with an increase in passive stiffness defined by the torque–angle curve of the elbow flexors at 48 h after eccentric exercise of the elbow flexors. Therefore, it is possible that a significant correlation between muscle hardness and CIR was due to the involvement of both passive tension and internal pressure increases.

As shown in Fig. 8A and E, a significant correlation was also found between MVIC force and muscle hardness (PM: r = − 0.565, *p* < 0.001, SE: r = − 0.516, *p* < 0.001) but the correlation coefficients were lower than that of RANG. Although the loss of force-generating ability is one of the best indirect markers of muscle damage^17^, muscle hardness reflected the increase in passive stiffness during measurement rather than loss of MIVC force. Since muscle hardness had a high correlation with contracting force^2^, if muscle hardness during force-generating was measured, the muscle hardness would be also decreased as MVIC force decreases.

There are several limitations in the present study. First, the participants of the study were only young men, and the sample size was not large, thus the results of the study may not reflect female and larger populations with different age groups. It has been reported that muscle stiffness is influenced by estradiol-b-17 and free testosterone^26^. It is necessary to investigate whether the findings of the present study are applicable for female participants. Secondly, the measurements within 24 h after exercise were not included in the present study, since the changes beyond 1 day post-exercise represent muscle damage better (the effects of muscle damage and fatigue are mixed at immediately after exercise)^27^. Furthermore, the participants performed ECC and CON alternately using both arms, thus a time difference (> 30 min) for the “immediately post-exercise” is caused for the measurements between the ECC and CON conditions. However, in order to understand the effects of hyperemia on muscle hardness changes, it would have been better to include the measurements soon after the eccentric versus concentric exercise. Cardio-pulmonary response in CON is larger than ECC^9^, so post-exercise vasodilatory action might have affected bilaterally immediately after exercise in the present study. Therefore, future study is warranted to consider the acute response of muscle hardness after ECC and CON by separating the two exercises such that each exercise is performed on a separate day. Moreover, the present study did not measure the passive tension during the elbow extension. Previous studies have shown an increase in passive tension in elbow extension after muscle damage^24,25^. It is important to confirm a direct relationship between muscle tension and hardness in the future studies. It is also necessary to investigate the mechanisms underpinning increased muscle hardness in muscle fiber and/or fascicle level. Lastly, the present study used strain elastography, but several studies ^28–30^ used shear wave elastography (SWE) to assess muscle stiffness changes after eccentric exercise. Regarding the SE and SWE according to a review article^31^, SWE mainly shows the shear speed changes in the longitudinal direction, which is an index of stiffness, while SE shows the strain change in the direction perpendicular (pressure) to the muscle tissue. No previous study has directly compared changes in SE and SWE after eccentric exercise, but the changes in both after static stretching have been shown to be similar^32^ and it has been pointed out that the two methods provide similar values. Lacourpaille et al.^30^ reported that shear modulus increased in the biceps brachii after eccentric exercise but not after concentric exercise, suggesting that muscle damage increases muscle stiffness. It is interesting to duplicate the present study using SWE. In addition, the strain distribution by SE depends on the depth and layer of the structure, and soft tissue can affect hardness^33^. Thus, in the SE and SWE assessments, mechanical properties should be considered as factors influencing their values in future studies. This may clarify the relationship between muscle hardness and muscle stiffness better.

In practical application, there is the possibility of using muscle hardness assessment as a muscle damage marker. Furthermore, there was a significant correlation between muscle hardness assessed by PM and SE. Therefore, a PM can evaluate muscle hardness in the same way as SE. PM is a space-saving and inexpensive device when compared to SE, and is convenient for sports and rehabilitation. The results of this study show the usefulness of muscle hardness evaluation using PM.

In conclusion, the results of the present study supported our hypotheses that muscle hardness would increase greater after ECC than CON, and PM and SE would indicate the same property in muscle hardness assessment. Changes in muscle hardness were significantly correlated with changes in indirect muscle damage markers such as MVIC force, CIR, RANG, and EANG. Both PM and SE had the highest correlation coefficient with RANG, suggesting that muscle hardness was most affected by the increase in passive stiffness. Muscle hardness evaluation using PM and SE is comparable and effective for post-exercise muscle conditioning as a new muscle damage marker.

## Data Availability

The datasets that were used and/or analysed in the current study are available from the corresponding author on reasonable request.
